# Assessing the role of central lymph node ratio in predicting recurrence in N1a low-to-intermediate risk papillary thyroid carcinoma

**DOI:** 10.3389/fendo.2023.1158826

**Published:** 2023-09-14

**Authors:** Teng Ma, Jian Cui, Peng Shi, Mei Liang, Wenxiao Song, Xueyan Zhang, Lulu Wang, Yafei Shi

**Affiliations:** ^1^ Department of Thyroid Surgery, Affiliated Hospital of Jining Medical University, Jining, Shandong, China; ^2^ Breast Disease Center, Affiliated Hospital of Qingdao University, Qingdao, Shandong, China; ^3^ Qingdao Medical College, Qingdao University, Qingdao, Shandong, China; ^4^ Department of Cardiovascular Surgery, Affiliated Hospital of Qingdao University, Qingdao, Shandong, China

**Keywords:** papillary thyroid carcinoma, lymph node ratio, structural recurrence, biochemical recurrence, total thyroidectomy

## Abstract

**Introduction:**

Lymph node metastasis in patients with papillary thyroid carcinoma (PTC) is associated with postoperative recurrence. Recently, most studies have focused on the evaluation of recurrence in patients with late-stage PTC, with limited data on those with early-stage PTC. We aimed to assess the relationship between lymph node ratio (LNR) and recurrence in low-to-intermediate-risk patients and validate its diagnostic efficiency in both structural (STR) and biochemical recurrence (BIR).

**Methods:**

Clinical data of patients with PTC diagnosed at the Affiliated Hospital of Jining Medical University were retrospectively collected. The optimal LNR cut-off values for disease-free survival (DFS) were determined using X-tile software. Predictors were validated using univariate and multivariate Cox regression analyses.

**Results:**

LNR had a higher diagnostic effectiveness than metastatic lymph nodes in patients with low-to-intermediate recurrence risk N1a PTC. The optimal LNR cutoff values for STR and BIR were 0.75 and 0.80, respectively. Multivariate Cox regression analysis showed that LNR≥0.75 and LNR≥0.80 were independent factors for STR and BIR, respectively. The 5-year DFS was 90.5% in the high LNR (≥0.75) and 96.8% in low LNR (<0.75) groups for STR. Regarding BIR, the 5-year DFS was 75.7% in the high LNR (≥0.80) and 86.9% in low LNR (<0.80) groups. The high and low LNR survival curves exhibited significant differences on the log-rank test.

**Conclusion:**

LNR was associated with recurrence in patients with low-to-intermediate recurrence risk N1a PTC. We recommend those with LNR≥0.75 require a comprehensive evaluation of lateral neck lymphadenopathy and consideration for lateral neck dissection and RAI treatment.

## 1 Introduction

Papillary thyroid carcinoma (PTC) accounts for approximately 90% of thyroid cancers and has a favorable prognosis, with a 10-year disease-specific mortality of approximately 4% (1, 2). However, the high incidence of its recurrence is nonnegligible. Although the majority of PTC recurrences are not fatal (3), they can cause significant suffering, particularly in countries such as China, where PTC is common (4).

To determine the risk of recurrence and establish a corresponding follow-up strategy and postoperative treatment plan, three risk categories have been recommended by the American Thyroid Association (ATA): low, intermediate, and high risk (5). Some studies have reported that radioactive iodine (RAI) ablation or prophylactic lateral neck dissection is required to reduce the risk of recurrence in high-risk patients with PTC (6–9). Nevertheless, patients in the low-to-intermediate-risk group also show a certain risk of recurrence (10). Patients with less than five metastatic lymph nodes (MLNs) are classified as low risk, but the number of MLNs varies according to the degree of lymph node (LN) dissection scope and pathological sampling extent (11). Some patients still exhibit a significant probability of recurrence under this system, even with fewer than five MLNs (12).

According to the American Joint Committee on Cancer (AJCC) 8th TNM classification of Differentiated Thyroid Carcinoma, N1a and N1b are categorized into the same stage (13). Therefore, the N staging of TNM classification is inadequate to assess the probability of recurrence in patients with positive LNs (14–16). Furthermore, it is unclear which patients with pN1a should undergo prophylactic lateral neck dissection to prevent lateral lymph node recurrence (17). To differentiate between “lower risk characteristics” and “higher risk characteristics” in these patients, a new risk stratification system is required in order to provide individualized therapy for each patient, reducing hazards and optimizing benefits.

The number of MLNs divided by the total number of lymph nodes (TLNs) is known as the lymph node ratio (LNR). This ratio has been utilized to assess oncological prognoses of solid tumors, including those of the lung (18), stomach (19), and colon (20). This study aimed to identify an optimal cutoff value for LNR and explore the relationship between LNR and patients with low-to-intermediate recurrence risk N1a PTC. Furthermore, we aimed to validate the value of LNR as an indicator of tumor structural recurrence (STR) and biochemical recurrence (BIR).

## 2 Materials and methods

### 2.1 Patient selection

Data from patients with PTC who were diagnosed at the Affiliated Hospital of Jining Medical University between December 2012 and December 2017 were retrospectively collected and analyzed. Of 2861 patients who underwent surgical treatment during the study period, 617 met the inclusion criteria.

The inclusion criteria were: having undergone total thyroidectomy and central lymph node dissection; being aged at least 18 years; being classified as pathological T1-3N1aM0 according to the AJCC 8th TNM Classification of Differentiated Thyroid Carcinoma system; being classified as low-to-intermediate risk according to the 2015 ATA management guidelines; having achieved excellent responses after initial surgery (suppressed thyroglobulin (Tg) < 0.2 ng/mL or thyroid-stimulating hormone [TSH]-stimulated Tg < 1 ng/mL and negative imaging); and not having undergone radioactive iodine (RAI) ablation postoperatively.

The exclusion criteria were postoperative persistent disease; other significant malignant tumors; serious medical record deficiency or loss during follow-up; and a TLN number less than 4.

This study was conducted in compliance with the 2013 revision of the Helsinki Declaration. The Ethics Committee of the Affiliated Hospital of Jining Medical University approved the study (2022C092). All participants provided written informed consent before participation.

### 2.2 Post-operative follow-up

All patients were followed up with and treated based on the ATA management guidelines (2009 or 2015 version) (5, 21). Outpatient reviews were recommended every 3–6 months for the first 2 years, and annually thereafter. Physical examination and thyroid ultrasonography were performed to evaluate the surgical area. Additionally, thyroid function was evaluated by testing serum thyroglobulin (Tg), anti-thyroglobulin antibodies (TgAb), and TSH levels. Data collected from medical records included age; sex; BMI; Hashimoto’s thyroiditis (HT) status; TNM staging; tumor number; tumor size; extrathyroidal extension (ETE); TLNs; MLNs; BRAF status; ultrasound and RAI scanning results; and Tg, TgAb, and TSH levels.

Based on evaluations at each follow-up visit after surgery, the disease outcomes of STR and BIR were diagnosed. STR was diagnosed based on cytological or histological proof, as well as clear results of ultrasound, computerized tomography (CT), RAI whole-body scans, or positron emission tomography-CT. BIR was defined as suppressed Tg levels of ≥1 ng/mL, TSH-stimulated Tg levels ≥10 ng/mL, or progressive rise in TgAb, without evidence of structural disease on imaging modality (22). Disease-free survival (DFS) was defined as the period without disease recurrence after initial treatment, including structural recurrence-free survival (STRFS) and biochemical recurrence-free survival (BIRFS). RAI ablation or follow-up was recommended for patients with BIR. However, RAI ablation, a second surgery, or further follow-up was recommended for patients with STR.

### 2.3 Statistical analysis

The Student’s t-test (normally distributed data) and Mann-Whitney test (non-normally distributed data) were used to compare continuous data. The chi-square test was used to compare categorical data. Continuous variables are presented as means ± standard deviations, whereas categorical variables are presented as numbers with percentages. The optimal cut-off values of LNR relevant to the STRFS and BIRFS were determined using X-tile software. The patients were allocated into groups according to the optimal cutoff values for LNR. The Kaplan–Meier method was used to evaluate survival rates, and the results were compared using the log-rank test. Predictors were validated using univariate and multivariate Cox regression analyses. Statistical significance was defined as a *p*-value of less than 0.05. X-tile and SPSS version 26.0 software were used for all statistical assessments.

## 3 Results

### 3.1 Patient characteristics and follow-up

This study included 617 patients (181 men and 436 women). **Table 1** presents the patient and tumor characteristics. The average age was 42.94 ± 14.35 years, and the average tumor size was 1.77 ± 0.75 cm. The ETE rate was 16.21%, the Hashimoto’s thyroiditisrate was 36.47%, and BRAF mutations rate was 52.19%. In addition, the median numbers of MLNs and TLNs were 4.17 ± 1.98 and 8.08 ± 3.02 in this study, respectively.

**Table 1:** Demographics and tumor characteristics.

	N	P(%)
Sex (Female)	436	70.66
Age,years (Mean ± SD)	42.94 ± 14.35	
Age, number (≥55)	173	28.04
Body Mass Index (mean ± SD)	25.47 ± 3.35	
Hashimoto’s thyroiditis, number	225	36.47
Bilateral tumors, number	54	8.75
Size, cm (mean ± SD)	1.77 ± 0.75	
Multifocality, number	110	17.83
Extrathyroidal extension	100	16.21
BRAF mutation, number	322	52.19
Metastasized lymph nodes (mean ± SD)	4.17 ± 1.98	
Total lymph nodes (mean ± SD)	8.18 ± 3.02	
Lymph node ratio	0.53 ± 0.23	
Follow-up time,months (mean ± SD)	69.69 ± 17.07	
Structural recurrence, number	31	5.02
Biochemical recurrence, number	94	15.24

**Figure 1:**
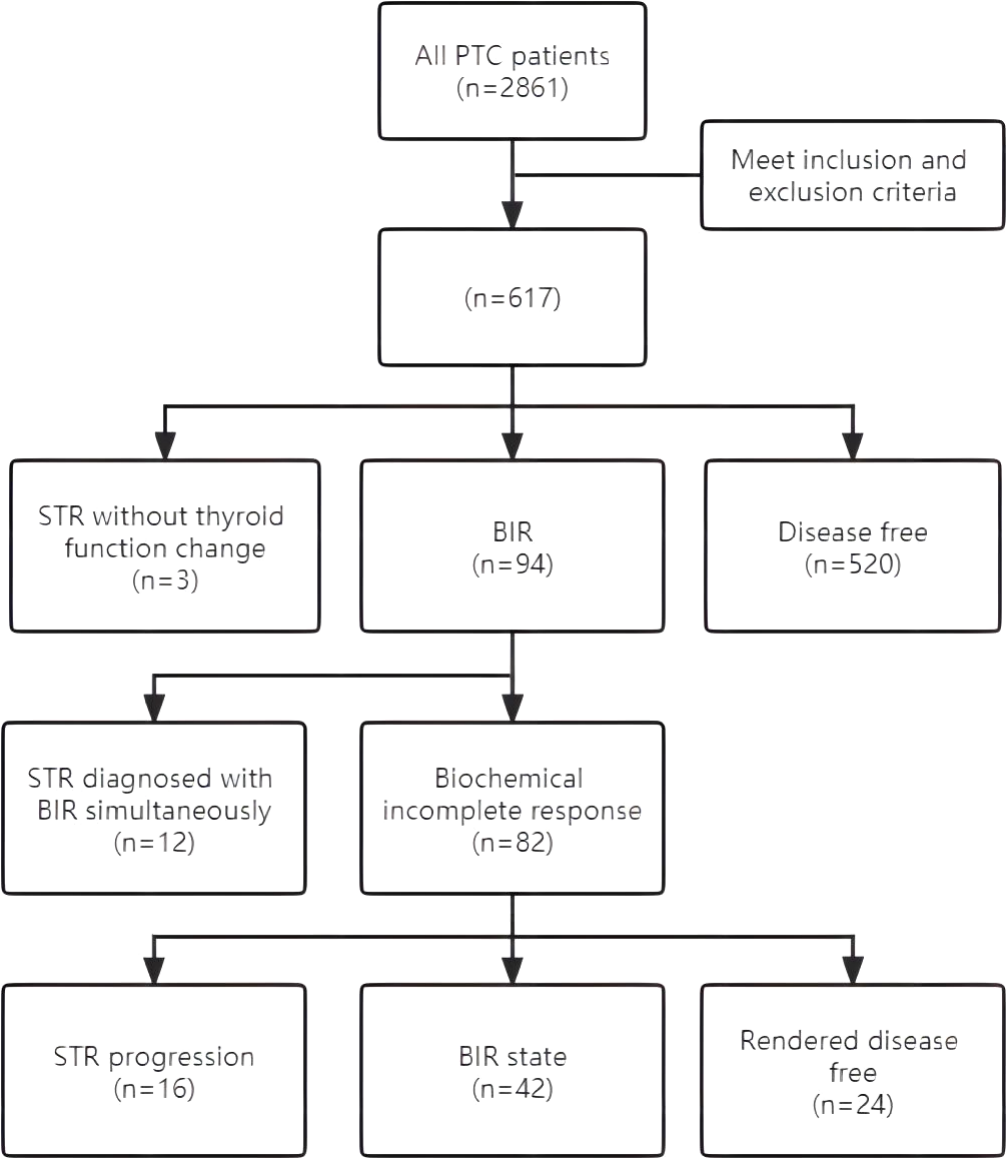
Flowchart for study sample.

Structural recurrence was found in 31 cases (5.02%): six in the initial thyroidectomy bed, seven in the cervical lymph nodes, and 18 in the lateral lymph nodes. Of these 31 patients, 24 were treated with secondary surgery and five with RAI ablation, while two chose to continue their follow-up (**Figure 1**).

Biochemical recurrence was found in 94 cases (15.24%): 37 patients were treated with RAI ablation and the others were followed up. Of these 94 patients, 12 were also diagnosed with STR. After diagnosis, the remaining 82 patients were monitored. The results showed that 16 patients developed STR in the following 1–35 months, including 10 patients who had undergone RAI. Twenty-four patients had a disease-free state at the last follow-up, 10 of whom had undergone RAI. Forty-two patients continued to have a biochemical incomplete response status, including 17 patients who had received RAI. Additional RAI ablation did not have a significant impact on the outcomes of the patients with BIR (**Table 2**).

**Table 2:** Clinical outcome of BIR patients.

	RAI	Non-RAI	Total	P-value
STR progression	10	6	16	
BIR keep	17	25	42	
Rendered disease-free	10	14	24	
Total	37	45	82	0.296

STR, structural recurrence; BIR, biochemical recurrence.

The median follow-up period for the group as a whole was 69.69 ± 17.07 months (range, 6–108 months). Nine patients died during follow-up due to PTC-unrelated events. At the last follow-up, 511 (82.82%) patients were alive and disease free.

### 3.2 Optimal LNR cut-off values

**Figure 2:**
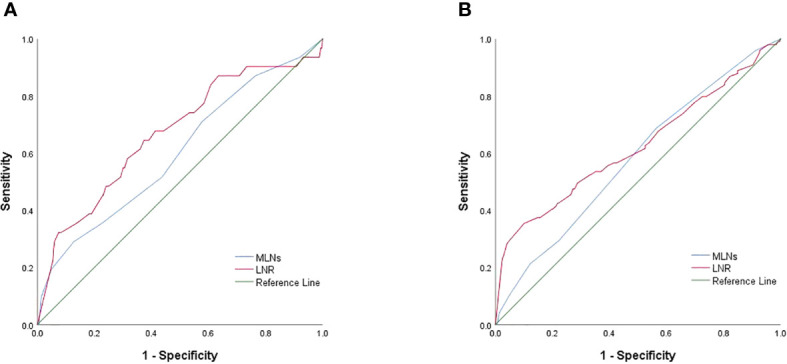
**(A)** ROC curves for LNR and MLNs in STR. **(B)** ROC curves for LNR and MLNs in BIR.

ROC curve analysis was performed to assess the diagnostic effectiveness of LNR and MLNs in STRFS and BIRFS. The results showed that the area under the curve (AUC) of the LNR was larger than that of the MLNs for both STRFS (0.669 versus 0.600) and BIRFS (0.558 versus 0.535) (**Figures 2A, B**). X-tile software was used to determine the ideal LNR cut-off value. The results indicated that, for STR and BIR, 0.75 and 0.80 were the optimal cut-off values, respectively. Baseline clinicopathological features were compared according to optimal LNR cut off values (**Tables 3** and **4**).

**Table 3:** Comparison of demographics and tumor characteristics according to optimal LNR cut off values of structural recurrence.

	LNR <0.75 N=469	HNR ≥0.75 N=148	P-value
Sex			0.044
Male	142	39	
Female	327	109	
Age (Mean ± SD)(y)	42.28 ± 14.12	45.01 ± 14.92	0.009
Age55			0.382
≥55	119	54	
<55	350	94	
BMI	25.54 ± 3.32	25.26 ± 3.47	0.598
HT			0.052
Absence	304	88	
Presence	165	60	
Bilaterral			0.483
Absence	422	141	
Presence	47	7	
Tumor size	1.784 ± 0.75	1.73 ± 0.75	0.557
Multifocality			0.201
Absence	383	124	
Presence	86	24	
Infiltration			0.478
Absence	398	119	
Presence	71	29	
BRAF mutation			0.000
Absence	228	67	
Presence	241	81	
MLN	3.83 ± 1.99	5.22 ± 1.54	0.000
TLN	8.84 ± 3.04	6.07 ± 1.72	0.006
Structural recurrence			0.044
Absence	452	134	
Presence	17	14	

STR, structural recurrence; BIR, biochemical recurrence; HT, Hashimoto’s thyroiditis;

MLN, metastatic lymph nodes; TLN, total lymph nodes.

**Table 4:** Comparison of demographics and tumor characteristics according to optimal LNR cut off values of biochemical recurrence.

	LNR <0.80 n=499	HNR ≥0.80 n=118	P-value
Sex			0.557
Male	149	32	
Female	350	86	
Age (Mean ± SD) (y)	42.40 ± 14.11	45.21 ± 15.17	0.056
Age55			0.004
≥55	127	46	
<55	372	72	
BMI	25.50 ± 3.32	25.38 ± 3.51	0.724
HT			0.867
Absence	320	72	
Presence	179	46	
Bilaterral			0.124
Absence	451	112	
Presence	48	6	
Tumor size	1.79 ± 0.74	1.70 ± 0.77	0.239
Multifocality			0.992
Absence	410	97	
Presence	89	21	
Infiltration			0.104
Absence	424	93	
Presence	75	25	
BRAF mutation			0.267
Absence	244	51	
Presence	255	67	
MLN	3.89 ± 1.20	5.34 ± 1.43	0.000
TLN	8.69 ± 3.07	6.02 ± 1.49	0.000
Biochemical recurrence			0.002
Absence	434	89	
Presence	65	29	

STR, structural recurrence; BIR, biochemical recurrence; HT, Hashimoto’s thyroiditis;

MLN, metastatic lymph nodes; TLN, total lymph nodes.

Considering STR, the high LNR (≥0.75) group had a higher recurrence rate than did the low LNR (<0.75) group (9.46% versus 3.63%, *p*=0.044). BIR was greater in the high LNR (≥0.80) group than in the low LNR (<0.80) group (24.58% versus 13.03%, *p*=0.002) group.

### 3.3 Risk factors for recurrence

Univariate analyses revealed that old age (≥55 years), tumor size, ETE, BRAF mutation, MLN number, and high LNR (≥0.75) were the main factors that influenced STRFS (*p*<0.10). Multivariate analyses showed that tumor size, ETE, BRAF mutation, MLN number, and high LNR (≥0.75) were independent influencing factors for STRFS (*p* < 0.05) (**Table 5**).

**Table 5:** Cox regression analysis of structural recurrence.

	Univariate Logistic Regression		Multivariate Logistic Regression	
	OR (95% CI)	P Value	OR (95% CI)	P Value
Sex	1.522(0.738-3.135)	0.255		
Age55	1.903(0.933-3.885)	0.077	1.564(0.761-3.214)	0.224
BMI	1.061(0.953-1.181)	0.281	NA	NA
HT	1.014(0.612-1.681)	0.957	NA	NA
Bilaterral	2.004(0.769-5.224)	0.155	NA	NA
Size	2.179(1.360-3.490)	0.001	1.798(1.134-2.851)	0.013
Multifocality	1.891(0.871-4.106)	0.107	NA	NA
Infiltration	4.660(2.296-9.457)	0.000	3.855(1.840-8.076)	0.000
BRAF mutation	2.216(1.020-4.815)	0.044	2.402(1.092-5.283)	0.029
MLN	1.232(1.039-1.461)	0.016	1.203(1.000-1.446)	0.050
TLN	0.979(0.869-1.103)	0.732	NA	NA
LNR	2.811(1.385-5.706)	0.004	2.130(1.018-4.456)	0.045

HT, Hashimoto’s thyroiditis; MLN, metastatic lymph nodes; TLN, total lymph nodes; LNR, lymph node ratio; NA, not applicable.

As regards BIRFS, univariate analyses demonstrated that old age (≥ 55 years), tumor size, multifocality, ETE, and high LNR (≥0.80) were relative influencing factors (*p*<0.10). Multivariate analyses showed that tumor size, ETE, and high LNR (≥0.80) were independent influencing factors (*p*<0.05) (**Table 6**).

**Table 6:** Cox regression analysis of biochemical recurrence.

	Univariate Logistic Regression		Multivariate Logistic Regression	
	OR (95% CI)	P Value	OR (95% CI)	P Value
Sex	1.386(0.910-2.111)	0.129	NA	NA
Age55	1.434(0.939-2.190)	0.095	1.241(0.805-1.912)	0.328
BMI	0.985(0.929-1.045)	0.620	NA	NA
HT	1.011(0.750-1.364)	0.940	NA	NA
Bilaterral	1.406(0.750-2.637)	0.288	NA	NA
Size	1.754(1.344-2.289)	0.000	1.519(1.162_1.986)	.002
Multifocality	1.535(0.959-2.456)	0.074	1.213(0.746-1.972)	0.436
Infiltration	3.680(2.427-5.582)	0.000	2.932(1.899-4.525)	0.000
BRAF mutation	1.226(0.815-1.845)	0.329	NA	NA
MLN	1.075(0.971-1.190)	0.166	NA	NA
TLN	1.012(0.946-1.082)	0.734	NA	NA
LNR	2.037(1.315-3.156)	0.001	1.953(1.254-3.043)	0.003

HT, Hashimoto’s thyroiditis; MLN, metastatic lymph nodes; TLN, total lymph nodes; LNR, lymph node ratio; NA, not applicable.

**Figure 3:**
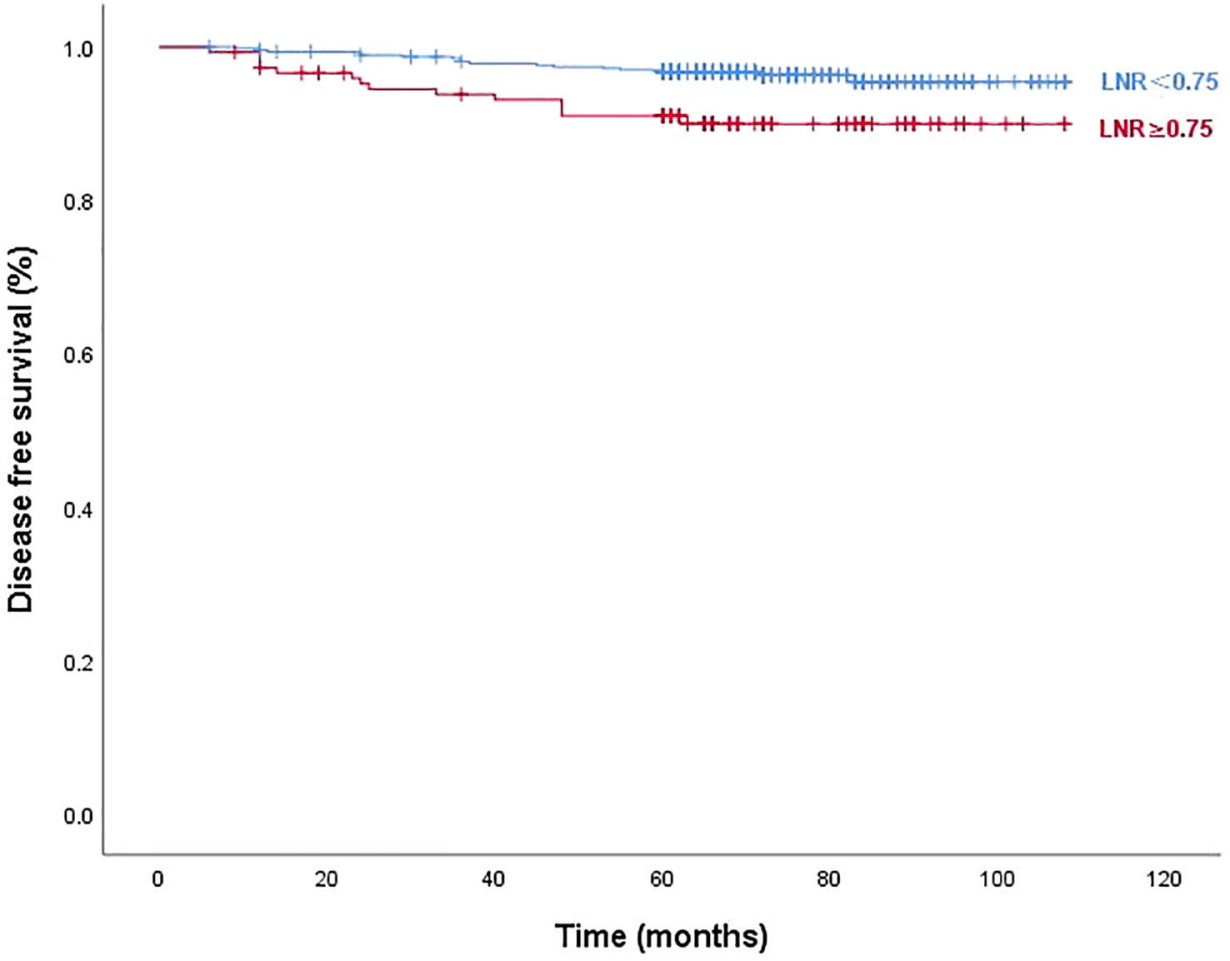
Disease-free survival curves according to optimal LNR cut off values of structural recurrence (log-rank p=0.003).

**Figure 4:**
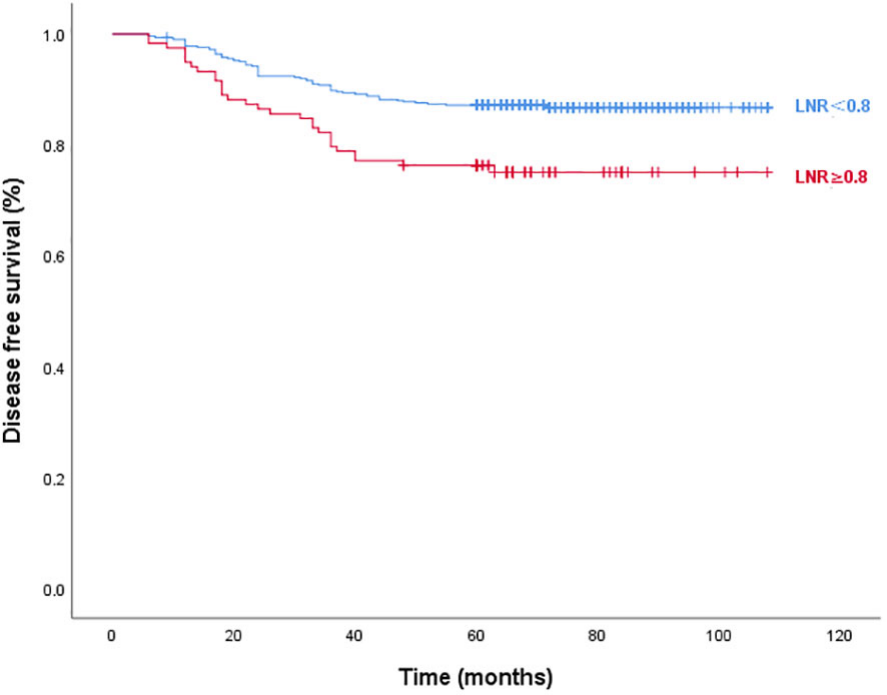
Disease-free survival curves according to optimal LNR cut off values of biochemical recurrence (log-rank p=0.001).

**Figures 3** and **4** show Kaplan–Meier curves grouped by the optimal LNR cut-off value. The high and low LNR survival curves exhibited significant differences in the log-rank test for both STRFS and BIRFS (*p*=0.003 and *p*=0.001, respectively). With regard to structural recurrence, the 5-year STRFS was 90.5% in the high LNR (≥0.75) and 96.8% in low LNR (<0.75) groups. However, in terms of biochemical recurrence, the 5-year BIRFS was 75.7% in the high LNR (≥0.80) and 86.9% in low LNR (<0.80) groups.

## 4 Discussion

PTC has a rather favorable prognosis, with a 10-year disease-specific mortality rate of approximately 4%. However, the high incidence of recurrence is nonnegligible. Although the majority of PTC recurrences are not deadly, they can be quite painful for patients.

Age, tumor size, pathological subtype, MLNs, ETE, and multifocality are risk variables that have been established as independent predictors of PTC recurrence. Various methods have been developed to determine the likelihood of recurrence based on these factors (23–25).

The 2015 ATA management guidelines classified recurrence risk as low, intermediate, and high, according to pathological features. These features included degree of residual lesion, tumor size, pathological subtype, envelope infiltration, vascular invasion, lymph node metastasis features, molecular pathological features, stimulated Tg level, and post-treatment whole-body scan (5). Local recurrence has been observed in 3–13% of patients with low-risk tumors, 21–36% of those with intermediate-risk tumors, and 68% of those with high-risk tumours (5). In this study, the median follow-up period was 69.69 ± 17.07 months, and total structural recurrence rate was 5.02%.

Lymph node metastasis in patients with PTC is associated with high recurrence rate. The number of MLNs varies with the degree of LNs dissection scope and pathological sampling ability. Some patients with fewer MLNs still have a high risk of recurrence. Are there other diagnostic indicators that, in combination with the existing ATA criteria, would be good predictors of recurrence in patients. According to the AJCC 8th TNM classification, N1a and N1b were categorized into the same stage. Therefore, the N staging of TNM classification is inadequate for assessing recurrence risk (26). Furthermore, it is unclear which patients with pN1a should undergo RAI ablation and which, prophylactic lateral neck dissection, to prevent recurrence. To differentiate between “lower risk characteristics” and “higher risk characteristics” in these patients, a new risk stratification strategy is required to provide individualized therapy for each patient, reducing hazards and optimizing benefits.

LNR has been widely used to assess oncological prognosis such as lungs, stomach, and colon cancer. In terms of PTC, after collecting data from 10, 955 patients, Schneider et al. (27) showed that LNR was associated with disease-related mortality. Parvathareddy et al. (28), in a retrospective study of 1407 cases, reported that TNM classification in combination with LNR had a stronger diagnostic capability for recurrence than did TNM alone. Lee et al. (29) claimed that the 2015 ATA risk stratification and 8th AJCC staging system provided greater predictive value for recurrence in patients with PTC once integrated with LNR. Kang et al. (30) reported that LNR was more accurate in predicting recurrence than N stage, after an analysis of a group of 307 patients with N1b PTC. Despite numerous findings on the use of LNR to assess PTC prognosis, no studies have assessed the clinical significance of LNR in patients with low-to-intermediate recurrence risk N1a PTC.

Our results showed that the diagnostic efficiency of LNR was higher than that of MLNs for STRFS (AUC of 0.669 versus 0.600). X-tile analysis showed that 0.75 was the optimal cut-off value for STR. Patients with LNRs of ≥0.75 had a significantly greater incidence of STR than did those with LNRs of <0.75 (9.46% versus 3.63%, *p*=0.044). Regarding STRFS, multivariate analyses showed that tumor size, ETE, BRAF mutation, MLN number, and high LNR (≥0.75) were independent influencing factors (*p*<0.05). Significant differences between the survival curves for high and low LNR were observed using the log-rank test (*p*=0.003). Five-year STRFS was 90.5% in the high and 96.8% in the low LNR groups. It is worth mentioning that the best cut-off value of LNR for predicting STR in this study was 0.8, which is higher than that reported in the relevant reports. We speculate that this is related to the different inclusion criteria, the patients included in this study were all low-to-intermediate risk patients (T1-3N1aM0), who had a better prognosis and a higher LNR cut-off value for the STR.

The majority of research evaluating long-term results frequently focuses on STR rather than BIR. A biochemical incomplete response is diagnosed based on increased serum levels of Tg or TgAb without any signs of structural abnormalities. In addition, if a patient achieved an excellent response after primary total thyroidectomy, this kind of biochemical incomplete response may be labelled BIR. According to the ATA, BIR is seen in 11–19% of low-risk, 2–22% of intermediate-risk, and 16–18% of high-risk patients (31, 32). However, the BIR rate of patients with N1a has not been assessed thus far. Our assessment revealed a 15.24% chance of BIR in patients with low-to-intermediate N1a. Since the long-term prognosis in these patients is not well understood, and some may develop structural illness, close follow-up is required. In this study, we also found that 0.80 was the optimal cutoff value for BIR. Patients with LNRs of ≥0.80 had significantly greater BIR incidences than did those with LNRs of <0.80 (9.46% vs. 3.63%, *p*=0.044). Multivariate analyses showed that tumor size, ETE, and high LNR (≥0.80) were independent factors influencing BIRFS (*p*<0.05). Significant differences between the survival curves for the high and low LNR groups were observed using the log-rank test (*p*=0.001). Five-year BIRFS was 75.7% for high LNR and 86.9% for low LNR. It is notable that, unlike the findings of other studies, performing RAI ablation after BIR had no significant effect on the progression to STR in our trial, which may have result from a high lymph node recurrence rate of STR in our cohort (33).

This retrospective study has several limitations that should be considered. Due to the possibility of selection bias in single-center studies, our findings may not be applicable to a larger population. Since we only included patients with pathologic N1a PTC, it might be challenging to extrapolate our findings to all patients with PTC. Furthermore, although certain patient data, such as histological subtypes, diameters of the biggest MLNs, extra-nodal extension and micrometastases of MLNs, did not appear in pathology reports 10 years ago, they were reported to have prognostic value (34–36). To overcome these restrictions, we intend to conduct prospective research in the future that also takes LN-related parameters into consideration. Our study also has a number of advantages. Every patient was diagnosed, treated, and followed up by the same medical team, in accordance with the same standardized procedure. In addition, our study had a longer follow-up period compared to that in similar studies. We also assessed factors influencing biochemical recurrence.

## 5 Conclusions

According to our findings, LNR was associated with recurrence in patients with N1a PTC. LNR≥0.75 and LNR≥0.80 were independent predictors of STRFS and BIRFS, respectively. We recommend those low-risk and intermediate-risk patients with a high LNR (≥0.75), even with limited MLNs, require a comprehensive evaluation of lateral neck lymphadenopathy and consideration for lateral neck dissection and RAI treatment. These findings may help physicians identify patients who are at risk and aid them in choosing the best postsurgical therapy and monitoring. The results of this research may help develop more effective staging standards.

## Data availability statement

The original contributions presented in the study are included in the article/supplementary material. Further inquiries can be directed to the corresponding author.

## Ethics statement

The experimental protocol was established, according to the ethical guidelines of the Helsinki Declaration and was approved by the Human Ethics Committee of Affiliated Hospital of Jining Medical University (2022C092).

## Author contributions

TM designed the study, analyzed the data and commented on the manuscript at all stages. WS and LW collected the data. JC,PS, XZ, ML revised the manuscript. YS provided the research direction. All authors contributed to the article and approved the submitted version.
